# Plasma calcitonin gene-related peptide and nitric oxide predict therapeutic response to amlodipine in pediatric primary hypertension

**DOI:** 10.3389/fphar.2024.1425863

**Published:** 2024-12-13

**Authors:** Hui Wang, Yao Lin, Yuting Wang, Chen Shen, Yaqi Li, Yang Liu, Lin Shi

**Affiliations:** ^1^ Department of Cardiology, Children’s Hospital, Capital Institute of Pediatrics, Beijing, China; ^2^ Children’s Hospital Capital Institute of Pediatrics, Chinese Academy of Medical Sciences & Peking Union Medical College, Beijing, China

**Keywords:** amlodipine, efficacy, biomarker, primary hypertension, children

## Abstract

**Background:**

There is a lack of suitable predictive markers for assessing the efficacy of amlodipine in treating children with primary hypertension. This study aimed to explore whether plasma calcitonin gene-related peptide (CGRP) and nitric oxide (NO) could predict the effectiveness of amlodipine in pediatric primary hypertension.

**Methods:**

This study enrolled 74 children and adolescents with primary hypertension who were prescribed amlodipine monotherapy, and after 4 weeks of treatment, they were divided into responders and non-responders according to blood pressure. Baseline data differences between the two groups were analyzed, followed by binary logistic regression to assess the correlation between significant variables and therapeutic efficacy. The receiver operating characteristic curve was used to evaluate the predictive efficacy, and the nomogram model was established to predict therapeutic response to amlodipine.

**Results:**

The responders exhibited lower body mass index, C-peptide and plasma CGRP levels, and higher NO levels compared to the non-responders (*p* < 0.05). Multivariable logistic analysis revealed that plasma CGRP and NO were independently associated with the therapeutic response to amlodipine, showing a higher predictive performance when used in combination (AUC: 0.814, 95% CI 0.714–0.914) with a predictive sensitivity of 86.5% and specificity of 70.1%. The nomogram model displayed good calibration, and the decision curve analysis indicated this model led to net benefits in a wide range of threshold probability.

**Conclusion:**

CGRP and NO may be valuable biomarkers for predicting amlodipine effectiveness in the treatment of pediatric primary hypertension, while the nomogram model indicates excellent predictive value.

## 1 Introduction

The increasing prevalence of obesity has led to a yearly rise in the incidence of hypertension among children and adolescents. A cohort study based on the China Health and Nutrition Survey showed a significant increase in the prevalence of hypertension in children and adolescents aged 7–17 from 8.5% in 1991 to 19.2% in 2015 (Ye et al., 2021), significantly exceeding the global prevalence range of 2%–5% (Samuels et al., 2019; Song et al., 2019). Notably, hypertension in childhood tends to persist into adulthood, thereby posing a major risk for cardiovascular morbidity and mortality in later life (Chen and Wang, 2008; Rapsomaniki et al., 2014; Samuels et al., 2019). Recent studies have also shown a significant association between adolescent hypertension and an increased risk of stroke in early adulthood (Fishman et al., 2023). Early treatment and proactive management of hypertension in children and adolescents are crucial.

Amlodipine is a well-established first-line antihypertensive drug for pediatric hypertension. As a long-acting calcium channel blocker, amlodipine inhibits calcium influx into smooth muscle cells, which is believed to be the primary mechanism leading to vascular relaxation. Notably, substantial interindividual variations in the therapeutic response to amlodipine are observed in the management of pediatric hypertension. Meta-analyses revealed that amlodipine achieved a significant decrease in both systolic and diastolic blood pressure (BP) in children with various medical conditions (Meyers and Siu, 2011). However, limited evidence suggests that only 30%–50% of patients achieve the target BP (Flynn et al., 2004; Parker et al., 2002; von Vigier et al., 2001). Therefore, identifying biomarkers to predict amlodipine effectiveness may help to improve the therapeutic effect. However, no relevant studies have been reported.

The calcitonin gene-related peptide (CGRP) is widely acknowledged as the most potent endogenous vasodilator, present in nearly all vascular nerve fibers and playing a crucial role in BP regulation (Al-Rubaiee et al., 2013). Studies have shown that CGRP acts with significant relaxant effects on all vessels and stimulates peripheral arterial vasodilation through nitric oxide (NO)-/endothelium-independent pathway in most vessels (Kumar et al., 2019; Kumar et al., 2022). NO is a free radical produced by L-arginine through nitric oxide synthase (NOS), which plays a crucial role in vasodilation and is a significant factor in the development of primary hypertension and other cardiovascular diseases (Bryan, 2022). Therefore, the study was conducted to investigate whether plasma CGRP and NO could successfully predict the effectiveness of amlodipine on pediatric primary hypertension, aiming to improve the effectiveness of individualized therapy with amlodipine.

## 2 Materials and methods

### 2.1 Study population

**FIGURE 1:**
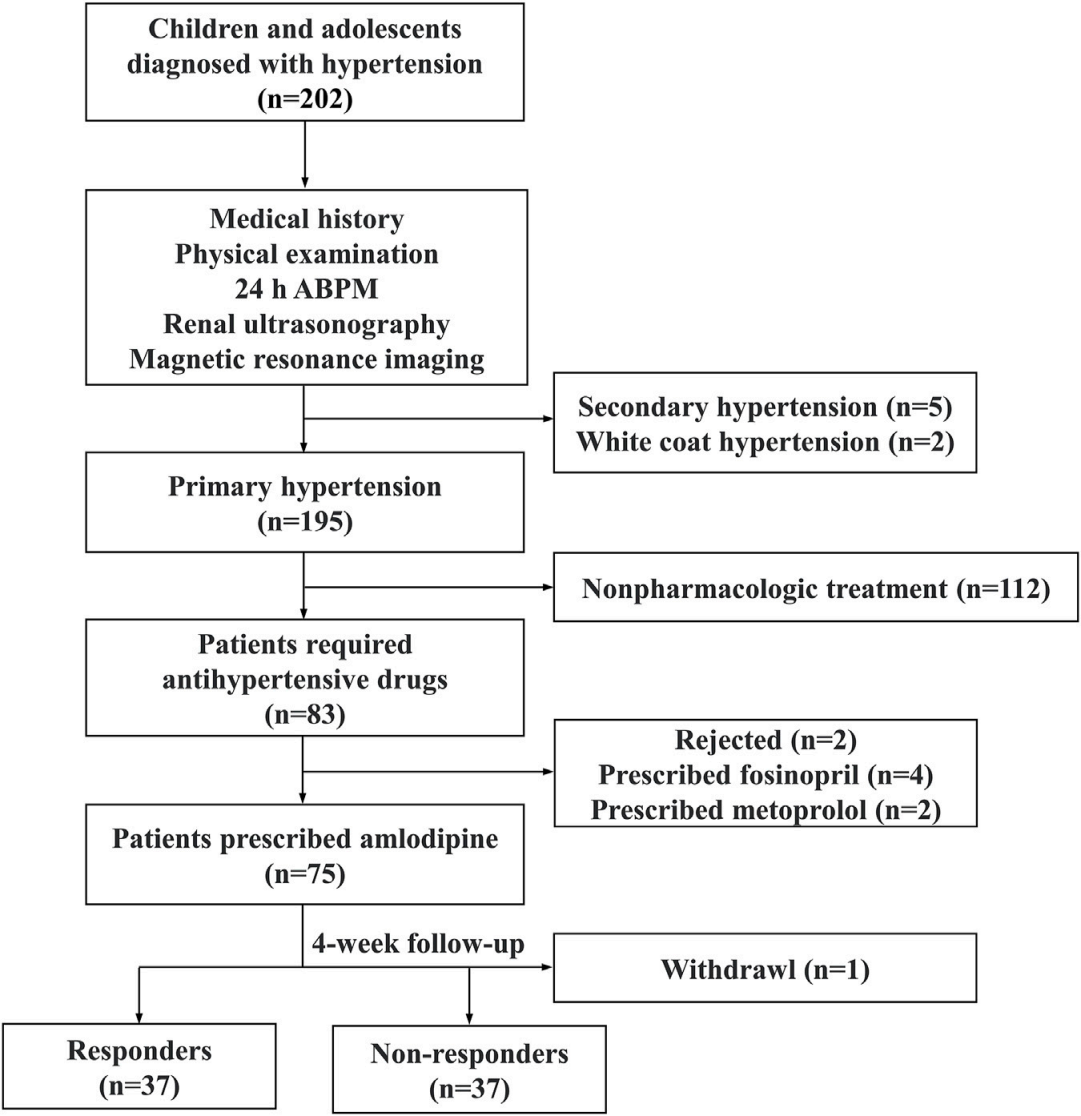
The study flowchart. ABPM ambulatory blood pressure monitoring.

Inclusion criteria for the participants included being aged 6–18 years, diagnosed with hypertension, and requiring antihypertensive medication. Patients with secondary hypertension, white coat hypertension, and primary hypertension receiving other antihypertensive medications were excluded. Secondary hypertension was evaluated by medical history, physical examination, 24-h urine vanillylmandelic acid, blood renin-angiotensin-aldosterone system levels, renal ultrasound, and craniocerebral magnetic resonance imaging. Moreover, 24-h ambulatory blood pressure monitoring was used to exclude white-coat hypertension (Bhatt et al., 2020; Flynn et al., 2017). Responders were defined as those with BP below the target systolic blood pressure (SBP) and diastolic blood pressure (DBP) after treatment, while others were defined as non-responders. The study flowchart is displayed in Figure 1.

### 2.2 Data collection, blood pressure measurement and diagnostic criteria

The demographic data of all subjects were collected. Laboratory test parameters of hypertensive patients were obtained from the electronic medical record system, including serum creatinine, uric acid, fasting blood glucose, alanine aminotransferase, aspartate aminotransferase, fasting insulin, C-peptide, total cholesterol, triglycerides, high-density lipoprotein-cholesterol, serum calcium, and urinary calcium; the estimated glomerular filtration rate was calculated as described elsewhere (de Simone et al., 2022). BP was measured using a mercury sphygmomanometer in all subjects and was graded according to the 2018 Chinese Guidelines for Prevention and Treatment of Hypertension (Joint Committee for Guideline Revision, 2019). Hypertension was diagnosed when SBP and/or DBP ≥95th percentile for age, sex, and height on at least 3 separate occasions. Stage 1 hypertension was defined as SBP and/or DBP ≥95th but <99th percentile +5 mmHg for age, sex, and height, while stage 2 was defined as SBP or DBP ≥99th percentile +5 mmHg for age, sex, and height.

### 2.3 Plasma calcitonin gene-related peptide and nitric oxide measurement

All subjects fasted for at least 8 h before 2 mL of cubital venous blood was collected in an EDTA anticoagulant tube in the morning. The collected blood samples were immediately subjected to centrifugation at 3,500 rpm for 15 min, and the upper plasma was carefully collected and stored at −80°C until further analysis. Plasma CGRP levels were measured using an enzyme-linked immunosorbent assay kit (Jiangsu Meimian Industrial Co., Ltd., Jiangsu, China), and plasma nitric oxide levels were evaluated using a nitric oxide assay kit (Nanjing Jiancheng Bioengineering Institute, Jiangsu, China), strictly following the manufacturer’s instructions. All the measurements were performed by a single-blinded researcher. All standards, controls, and samples were assayed simultaneously in duplicate.

### 2.4 Treatment and follow-up protocols

Patients with primary hypertension were initially prescribed 5 mg of amlodipine daily, targeting a BP at the 95th percentile for age, sex, and height. A physician evaluated patients weekly for BP reduction and adverse events, and if necessary, the dosage was adjusted starting from the second week of treatment, with a maximum daily dose of 10 mg. According to Flynn’s study, amlodipine treatment can achieve stable BP levels after 4 weeks (Flynn et al., 2004). Therefore, the last follow-up for BP measurement was scheduled after 4 weeks of treatment. For patients receiving the maximum dose but still not achieving the target BP, replacement or combined antihypertensive medications were considered. It is important to highlight that all participants underwent a standardized lifestyle intervention, including a low-sodium and low-fat diet, heightened aerobic exercise and weight reduction, under the guidance and supervision of the certified nutritionist and pediatrician, and demonstrated high adherence throughout the treatment period.

### 2.5 Statistical analyses

All statistical analyses were performed using SPSS 23.0, GraphPad Prism 8 software and R version 4.2.2 for Windows. Data normality was tested by the Kolmogorov-Smirnov test. Parametric continuous data were expressed as mean ± standard deviation (SD) and compared with the student t-test between groups. Non-parametric data were presented as median (the interquartile range) and analyzed by the Mann-Whitney test. The Chi-squared test was used to compare categorical variables. Variables with *P* less than 0.05 and demographic variables were included in a binary logistic regression model. The receiver operating characteristic (ROC) curve and areas under the curve (AUCs) were used to evaluate the accuracy of the selected major factors in predicting amlodipine efficacy. The sensitivity and specificity were highest at the points of ROC curves closest to the top left corner. Furthermore, a pairwise comparison between the two AUCs was performed using the DeLong method (Delong et al., 1988). A nomogram was developed based on multivariate regression analysis to facilitate clinical interpretation, the discriminability of the prognostic nomogram was evaluated using the calibration curve, and decision curve analysis (DCA) was performed to test the clinical applicability of the model. *p* < 0.05 was considered statistically significant.

## 3 Results

### 3.1 Patient characteristics

Totally 74 children with primary hypertension were included in the study, with 37 identified as responders and 37 categorized as non-responders. There were no significant differences in age, gender, height, baseline SBP and DBP, aminotransferase, serum lipid and calcium between the two groups (Table 1). However, responders exhibited lower body mass index (BMI), fasting C-peptide and plasma CGRP levels compared to non-responders (all *p* < 0.05), while plasma NO levels were higher (*p* < 0.05).

**TABLE 1:** Comparison of baseline characteristics between responders and non-responders.

Characteristic	Responders (*n* = 37)	Non-responders (*n* = 37)	*P*-value
Male (n, %)	30 (81.1)	30 (81.1)	1.000^a^
Age (years)	13.0 (2.0)	13.0 (2.0)	0.493^b^
Height (m)	1.71 (0.15)	1.71 (0.13)	0.669^b^
Weight (kg)	81.36 ± 19.93	88.57 ± 17.73	0.104^c^
BMI (kg/m^2^)	28.27 ± 4.50	30.64 ± 4.95	0.035^c^
SBP (mmHg)	140.0 (13.0)	144.0 (17.5)	0.128^b^
DBP (mmHg)	80.0 (10.5)	82.0 (14.0)	0.244^b^
Family history (n, %)	21 (56.8)	26 (70.3)	0.227^a^
Stage 2 (n, %)	32 (86.5)	34 (91.9)	0.708^a^
Dosage (mg/kg)	0.06 (0.03)	0.07 (0.03)	0.144^b^
Total cholesterol (mmol/L)	3.90 ± 0.67	3.92 ± 0.91	0.953^c^
Triglyceride (mmol/L)	1.14 (0.91)	1.24 (0.80)	0.837^b^
HDL-C (mmol/L)	1.08 ± 0.18	1.05 ± 0.19	0.502^c^
LDL-C (mmol/L)	2.62 ± 0.59	2.65 ± 0.96	0.876^c^
FBG (mmol/L)	4.66 (0.51)	4.54 (0.80)	0.991^b^
ALT (U/L)	19.80 (9.45)	23.40 (15.70)	0.254^b^
AST (U/L)	18.30 (8.00)	18.80 (7.50)	0.224^b^
Fasting insulin (uIU/mL)	19.50 (11.45)	22.90 (21.70)	0.056^b^
Fasting C-peptide (ng/mL)	2.79 (1.24)	3.42 (1.53)	0.042^b^
CGRP (ng/L)	56.67 ± 17.55	80.19 ± 25.71	<0.001^c^
NO (μmol/L)	42.72 (33.40)	33.91 (33.74)	0.041^b^
Serum calcium (mmol/L)	2.44 (0.08)	2.41 (0.12)	0.176^b^
Urine calcium (mmol/L)	1.40 (1.61)	1.58 (1.78)	0.372^b^
Serum creatinine (µmol/L)	55.20 ± 12.55	52.06 ± 14.01	0.313^c^
Serum uric acid (mmol/L)	448.65 ± 102.86	485.08 ± 102.61	0.132^c^
eGFR (ml/min/1.73 m^2^)	110.15 ± 18.16	115.28 ± 18.88	0.237^c^

BMI, body mass index; SBP, systolic blood pressure; DBP, diastolic blood pressure; HDL-C, high-density lipoprotein cholesterol; LDL-C, low-density lipoprotein cholesterol; FBG, fasting blood glucose; ALT, alanine aminotransferase; AST, aspartate aminotransferase; CGRP, calcitonin gene-related peptide; NO, nitric oxide; eGFR, estimated glomerular filtration rate. The continuous variables were presented as mean ± standard deviation or median (interquartile range).

^a^ chi-square test.

^b^ Mann–Whitney U test.

^c^ Student T test.

### 3.2 Predictive value of plasma calcitonin gene-related peptide and nitric oxide

**FIGURE 2:**
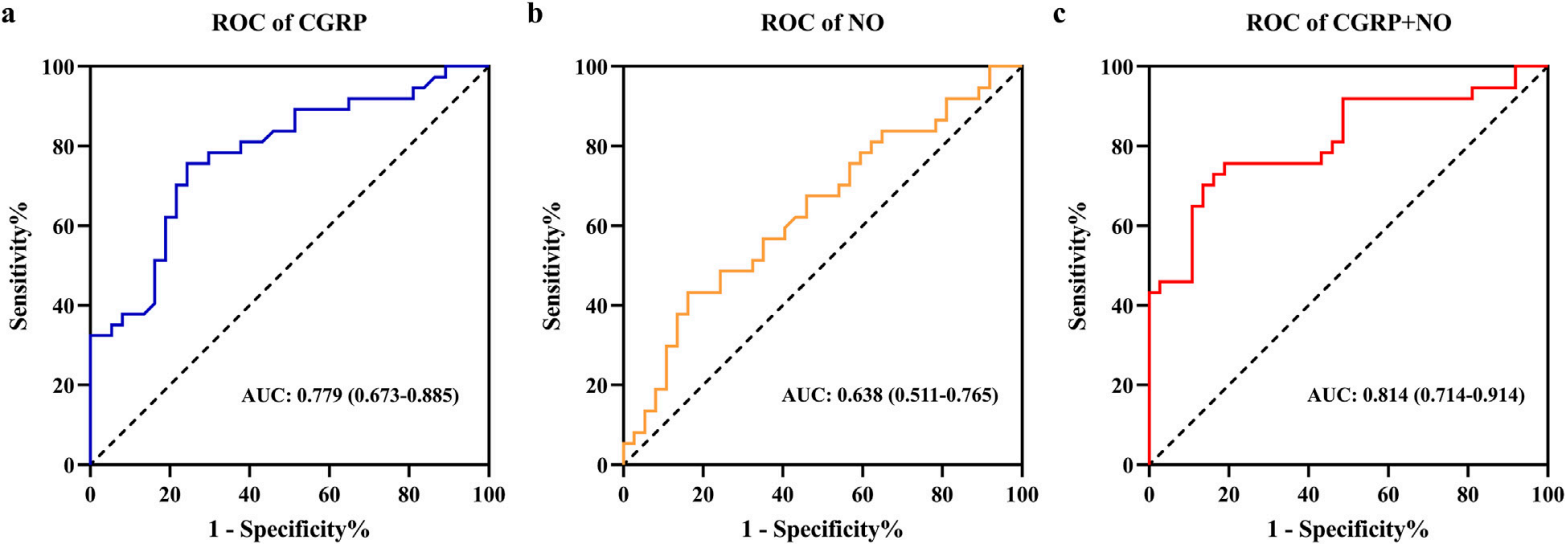
Receiver operating characteristic curves analysis of plasma CGRP and NO levels for predicting amlodipine therapeutic efficacy. **(A)** ROC of CGRP. **(B)** ROC of NO. **(C)** ROC of CGRP + NO. CGRP, calcitonin gene-related peptide; NO, nitric oxide.

Factors with significant differences were included in binary logistic regression analysis. After adjusting for age and gender, results indicated that plasma CGRP (odds ratio (OR) = 0.944, 95% confidence interval (CI): 0.912–0.978, *p* = 0.001) and NO (OR = 1.027, 95%CI: 1.001–1.053, *p* = 0.041) were independently associated with amlodipine responsiveness (Table 2). Furthermore, The AUCs of plasma CGRP and NO in predicting the response to amlodipine were 0.779 (95% CI 0.673–0.885) and 0.638 (95% CI 0.511–0.765), with the corresponding cutoff values of 67.54 ng/L and 23.80 μmol/L, respectively (Table 3; Figure 2). To further improve the predictive values, a model was constructed based on logistic regression as follows: Logit(P) = 2.899 - 0.058 × CGRP + 0.024 × NO. The AUC for the combined prediction model was 0.814 (95% CI 0.714–0.914, Figure 2) with the optimum prediction cut-off value 0.484, producing a predictive sensitivity and specificity of 86.5% and 70.1%. Pairwise comparison indicated that the combined model exhibited better predictive performance (*p* < 0.05, Table 3).

**TABLE 2:** Multivariate logistic regression of amlodipine therapeutic efficacy.

Variables	B	SE	Wald	OR	95% CI	*P*-value
Age	0.089	0.148	0.362	1.093	0.818–1.460	0.547
Male	−0.036	0.757	0.002	0.965	0.219–4.251	0.962
BMI	−0.082	0.081	1.027	0.921	0.787–1.079	0.311
C-peptide	0.001	0.343	0.000	1.001	0.511–1.961	0.997
CGRP	−0.057	0.018	10.512	0.944	0.912–0.978	0.001
NO	0.026	0.013	4.193	1.027	1.001–1.053	0.041
Constant	4.048	2.360	2.941	57.297	—	0.086

SE, standard error; OR, odd ratio; CI, confidence interval; BMI, body mass index; CGRP, calcitonin gene-related peptide; NO, nitric oxide.

**TABLE 3:** The cut-off value of predictor variables predicting amlodipine efficacy in pediatric primary hypertension.

Variables	AUC	95% CI	Cut-off value	Sensitivity	Specificity	*P*-value
CGRP	0.779	0.673–0.885	≤67.54 ng/L	75.7%	75.7%	0.008
NO	0.638	0.511–0.765	≥23.80 μmol/L	83.8%	43.2%	0.037
Combined Prediction	0.814	0.714–0.914	≥0.484	86.5%	70.1%	—

ROC, receiver operating characteristic; AUC, the area under the ROC, curve; CI, confidence interval; CGRP, calcitonin gene-related peptide; NO, nitric oxide. The P-value indicates comparisons with the combined model.

### 3.3 Nomogram model establishment and clinical application evaluation

**FIGURE 3:**
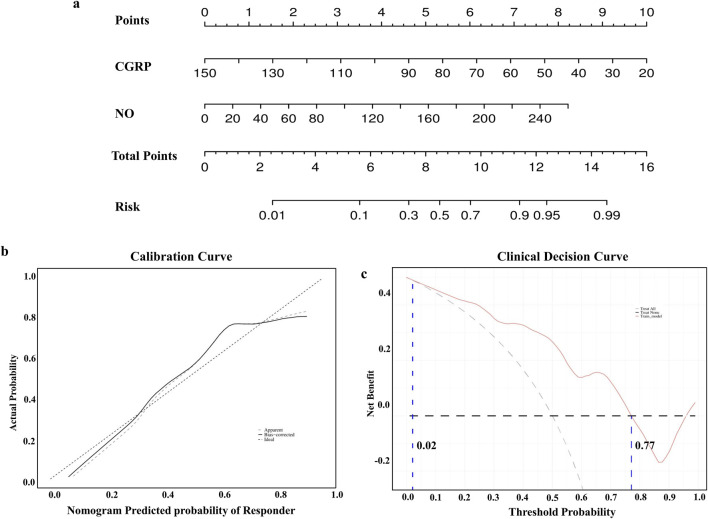
Construction of the nomogram model. **(A)** Nomogram model predicting the therapeutic efficacy of amlodipine. **(B)** Calibration curves for nomogram model predicting the therapeutic efficacy of amlodipine. **(C)** Decision curve analysis showing higher net benefit with nomogram model. CGRP, calcitonin gene-related peptide; NO, nitric oxide.

To enhance clinical interpretation, a nomogram model was developed to predict amlodipine treatment efficacy based on binary logistic regression analysis (Figure 3A). The model revealed that the effective rate of amlodipine increased with the reduction in CGRP and the elevation of NO levels before treatment. The calibration curve confirmed that the predicted results of the nomogram were close to the actual observations (Figure 3B). Furthermore, the DCA curve showed that if the threshold probability is between 2%–77%, the application of this nomogram to predict the efficacy of amlodipine would add up to 0.49 net benefit (Figure 3C). These results suggested the favorable clinical applicability of our nomogram model.

## 4 Discussion

In this study, we followed 74 children with primary hypertension treated with amlodipine, and after 1 month of treatment, 50% of them achieved the target BP. There were statistical differences in plasma CGRP and NO between the responders and non-responders, and a combined prediction model of the two factors could accurately predict the efficacy of amlodipine, with the AUC was 0.814 (95% CI 0.714–0.914), yielding the sensitivity and specificity of 86.5% and 70.1%, respectively. Furthermore, a nomogram model was developed to predict the response to amlodipine, facilitating decision-making for clinicians and displaying good clinical practicability.

Primary hypertension is the leading form of hypertension in children and adolescents, which is thought to be associated with impaired vasodilatation due to an imbalance between vasoconstrictors and vasodilators. Therefore, pharmacological treatment focuses on this process (Ambrosino et al., 2022; Ye et al., 2019). Amlodipine, the only calcium channel blocker recommended by the Food and Drug Administration for pediatric hypertension treatment, has been widely reported for its efficacy and safety (Flynn, 2005). However, upon reviewing the existing evidence, considerable variability in the antihypertensive response to amlodipine is observed among children with hypertension, with an effective rate of less than 50% (Meyers and Siu, 2011). Therefore, the identification of convenient, rapid, and affordable indicators that can accurately predict the effectiveness of amlodipine before treatment would greatly enhance the control rate of amlodipine therapy in pediatric hypertension patients, which is indispensable to implement individualized treatment strategies. Nevertheless, no relevant studies have been reported.

CGRP, a 37-amino acid neuropeptide produced by the calcitonin gene, is currently recognized as the most potent vasodilator, which exerts vasodilation through the activation of the CGRP receptors on smooth muscle cells. The plasma levels of CGRP were observed significantly decreased in patients with primary hypertension and spontaneous hypertensive rat models (Li et al., 2009; Smillie and Brain, 2011). Previous studies have shown a significant correlation between plasma CGRP levels and vasodilatory effects in children with vasovagal syncope (VVS). Notably, VVS children with higher CGRP levels tend to exhibit a more favorable therapeutic response to the vasoconstrictor drug midodrine hydrochloride (Li et al., 2022). In this study, we found that lower baseline CGRP levels could predict a better response to amlodipine treatment in pediatric hypertension. Studies have shown that CGRP release is calcium channel dependent (Chichorro et al., 2024; Smillie and Brain, 2011). A clinical study examining the efficacy of a traditional Chinese medicine prescription for primary hypertension treatment revealed a significant increase in plasma CGRP levels following 12 weeks of amlodipine treatment (Zhong et al., 2010). Intravenous administration of exogenous CGRP has also shown notable antihypertensive effects in hypertensive rats and human volunteers (DiPette et al., 1989; Gennari and Fischer, 1985). Furthermore, it has been found that amlodipine enhances the sensitivity of the CGRP receptor (Hong et al., 1997). Therefore, children with hypertension may benefit from amlodipine treatment due to its ability to enhance the sensitivity of the CGRP receptor and increase plasma CGRP levels, resulting in greater blood pressure reduction among those with lower baseline CGRP levels. These findings suggest that measuring plasma CGRP can serve as an early predictive indicator of the effectiveness of amlodipine in pediatric primary hypertension, which may also be suitable for the guidance of antihypertensive treatment in children with secondary hypertension.

NO is a crucial signaling molecule in endothelial function. Subsequently, it acts on vascular smooth muscle cells to activate guanylate cyclase and induce upregulation of cyclic guanosine monophosphate (cGMP) expression, which indirectly activates ATP-sensitive K+ channels and participates in the regulation of vascular relaxation, inhibition of smooth muscle cell proliferation, and inhibition of platelet aggregation. Studies have shown that hypertensive adolescents exhibit significantly lower plasma NO levels compared to normotensive adolescents of the same age (Aflyatumova et al., 2018). *In vitro* studies have shown that amlodipine enhances NO production and prolongs its half-life in endothelial cells, thereby improving NO bioavailability (Berkels et al., 2004; Sharma et al., 2011). We observed that plasma NO levels are higher in responders than in non-responders, and NO can predict the antihypertensive response to amlodipine, which may be attributed to elevated BMI, insulin, and C-peptide levels in non-responders, indicating the presence of insulin resistance, which mediates endothelial dysfunction leading to reduced NO synthesis and utilization (Clayton et al., 2023). Previous research has also indicated that lower levels of NO may be associated with more severe endothelial dysfunction (da Silva et al., 2021), and these patients may exhibit better responses to prolonged treatment and improved endothelial function. However, our study only followed up on the efficacy of amlodipine for 1 month, and further investigation is underway to assess long-term efficacy.

Furthermore, the nomogram model constructed by combining CGRP and NO demonstrated superior predictive performance and clinical practicability. Studies have indicated that CGRP is present in nearly all vascular nerve fibers and exerts a significant relaxant effect on blood vessels. In most blood vessels, CGRP binds to CGRP receptors on vascular smooth muscle cells, activating adenylyl cyclase to synthesize cyclic adenosine monophosphate (cAMP), which then induces ATP-sensitive K+ channel activation through protein kinase A, resulting in smooth muscle relaxation and vasodilation (Kumar et al., 2022; Younis et al., 2024). Furthermore, animal experiment has shown that the CGRP receptor-mediated contribution to external Ca^2+^ concentration-induced vasorelaxations predominantly involve an endothelium-dependent pathway (Carlton-Carew et al., 2024). Within vascular endothelial cells, CGRP can facilitate the activation of endothelial NOS (eNOS) through a cAMP signaling cascade, thereby promoting the synthesis and release of NO, but only in the presence of an intact endothelium (Kumar et al., 2022; Brabenec et al., 2022). Consequently, amlodipine treatment promotes the release of CGRP and enhances its receptor sensitivity, thereby facilitating the relaxation of vascular smooth muscle. Furthermore, it further stimulates eNOS to facilitate the production and release of NO, which mediates vasodilation in hypertensive children with intact endothelial function, ultimately leading to a more significant reduction in blood pressure. Further investigation into the underlying mechanisms of CGRP and NO involved in the antihypertensive effect of amlodipine is warranted.

To our knowledge, this is the first study to investigate predictive markers for amlodipine efficacy in pediatric hypertension and to develop a nomogram model for individualized treatment. However, the limitations of the current study should be acknowledged. First, the plasma CGRP and NO levels after treatment were not measured. Second, this is a single-center study with a small sample size that cannot represent the entire pediatric population and has not been externally validated, which may lead to certain extrapolation limitations of our results. In the future, multi-center, large-sample prospective studies are necessary to validate the reliability of the nomogram model, provide clinical individualized medication guidance for pediatricians, and enhance amlodipine therapeutic efficacy on primary hypertension in children.

## 5 Conclusion

Our findings suggest that plasma CGRP and NO may serve as valuable biomarkers for the effectiveness of amlodipine treatment in children with primary hypertension, and the newly developed nomogram model indicates good prediction accuracy and clinical application.

## Data Availability

The raw data supporting the conclusions of this article will be made available by the authors, without undue reservation.
